# Ectopic Expression of PII Induces Stomatal Closure in *Lotus japonicus*

**DOI:** 10.3389/fpls.2017.01299

**Published:** 2017-07-25

**Authors:** Aurora Parlati, Vladimir T. Valkov, Enrica D'Apuzzo, Ludovico M. Alves, Angelo Petrozza, Stephan Summerer, Alex Costa, Francesco Cellini, Alain Vavasseur, Maurizio Chiurazzi

**Affiliations:** ^1^Department of Biology, Agriculture and Food Sciences, Institute of Biosciences and Bioresources, Consiglio Nazionale delle Ricerche Napoli, Italy; ^2^ALSIA Centro Ricerche Metapontum Agrobios Metaponto, Italy; ^3^Department of Bioscience, University of Milan Milan, Italy; ^4^Department of Physics, Institute of Biophysics, Consiglio Nazionale delle Ricerche Milan, Italy; ^5^Unitè Mixte de Reserche 6191 Centre National de la Reserche Scientifique, Institute de Biologie Environnementales – Commissariat à l'Energie Atomique-Universitè Aix-Marseille II St. Paul Lez Durance, France

**Keywords:** PII, overexpression, stomatal movement, nitric oxide, nitrate reductase, *L. japonicus*

## Abstract

The PII protein in plants has been associated to many different tissue specialized roles concerning the Nitrogen assimilation pathways. We report here the further characterization of *L. japonicus* transgenic lines overexpressing the PII protein encoded by the *LjGLB1* gene that is strongly expressed in the guard cells of Lotus plants. Consistently with a putative role played by PII in that specific cellular context we have observed an alteration of the patterns of stomatal movement in the overexpressing plants. An increased stomatal closure is measured in epidermal peels from detached leaves of normally watered overexpressing plants when compared to wild type plants and this effect was by-passed by Abscisic Acid application. The biochemical characterization of the transgenic lines indicates an increased rate of the Nitric Oxide biosynthetic route, associated to an induced Nitrate Reductase activity. The phenotypic characterization is completed by measures of the photosynthetic potential in plants grown under greenhouse conditions, which reveal a higher stress index of the PII overexpressing plants.

## Introduction

PII proteins belong to one of the most conserved families of signaling proteins in nature described in almost all free-living bacteria, in nitrogen-fixing archaea and in chloroplast of red algae and green plants (Forchhammer, 2004; Huergo et al., 2013). Many bacteria and archaea have multiple PII proteins (encoded by *glnB, glnK*, or *nifI* genes), whereas a single chloroplast protein is found in green plants and green algae (encoded by the nuclear *GLB1* gene). Recently, an even larger superfamily of trimeric sensory PII-like proteins, based on structural similarities deduced from crystal structures has been proposed (Forchhammer and Luddecke, 2016). Canonical PII proteins play a central role on the coordination of carbon/nitrogen balance by controlling many different target proteins including enzymes, transcription factors and membrane transporters, via binding of adenyl nucleotides and 2-oxo-glutarate (2-OG). PII multitasking regulators are small proteins characterized by a trimeric structure with the most conserved mechanism of action occurring through binding at the clefts between the monomeric subunits of the molecules transmitting information; this is perceived via conformational changes of the highly flexible T-loop structure and transmission of the resulting state to many different targets through protein interactions. PII binding capacity for 2-OG (mostly in conjunction with ATP), a key intermediate of the Krebs. cycle reflects the cellular nitrogen status (low N/high 2-OG; high N/low 2-OG), whereas ATP and ADP competition for binding to PII mediates the sensing of cellular energy charge (Forchhammer and Hedler, 1997; Xu et al., 1998; Gerhardt et al., 2012; Oliveira et al., 2015). In addition to this mechanism where the different relative cellular concentration of the binding molecules controls their competitive interactions with the PII active sites, an ATPase activity reported in *E. coli, A. brasilense* and *A. thaliana* with a potential impact on PII activity has been proposed (Radchenko et al., 2013), but the physiological relevance of this enzymatic activity has been recently questioned (Luddecke and Forchhammer, 2015). Moreover, a superposed level of PII regulation is represented by post-translational modifications, which may take the form of phosphorylation (cyanobacteria), uridylylation (proteobacteria), or adenylylation (actinobacteria) that strongly affect the capacity of PII to interact with a variety of effector molecules (Merrick, 2015).

Despite the central role played as an integrator of C and N cellular metabolism in many prokaryotes, in plants PII evolved a secondary, tissue specialized role on the control of N assimilation signaling pathways. A high degree of functional similarity has been reported for PII of higher plants in particular with cyanobacterial PII proteins (Beez et al., 2009), although a post-translational modification has never been found (Smith et al., 2004). PII functions in higher plants have been mainly studied through functional characterization of *Arabidopsis thaliana* mutants, which demonstrated its involvement in the control of the Arg biosynthetic pathway, through interaction with N-Acetyl Glutamate Kinase (NAGK) to reduce feedback inhibition by high Arg concentrations (Sugiyama et al., 2004; Chen et al., 2006; Ferrario-Mèry et al., 2006). In addition, *A. thaliana* null mutants display alterations of the chloroplasts nitrite uptake (Ferrario-Mèry et al., 2008), whereas the reported major fluctuation of the *GLB1* transcript during the *A. thaliana* seed maturation (Uhrig et al., 2009) has been associated to a crucial role played by PII in the fine tuning of fatty acid biosynthesis and partitioning in Arabidopsis seeds (Baud et al., 2010). More recently, the striking observation that in the unicellular green algae *Chlamidomonas reinhardtii* the complex PII-NAGK could be observed only in the presence of glutamine, led to the identification of a glutamine sensing pathway acting via binding at the C-terminal sequence, which is highly conserved in almost all plant PII proteins with the exception of *A. thaliana* and the whole *Brassicaceae* family, where a short deletion is present (Chellamuthu et al., 2014).

In higher plants the control of water loss and CO_2_ uptake for photosynthetic performances occurs via the regulation of the size of the stomatal pore and the hormone largely governing the physiology of guard cells responses is the Abscisic Acid (ABA). Stomatal aperture is controlled through a balance between the light-promoting opening and the ABA mediated closure and opening inhibition. Nitrogen (N) might directly or indirectly influences the photosynthetic performances being an important component for the synthesis of pigments and photosynthetic enzymes. Nitrate uptake in the guard cells plays a positive role on stomatal opening (Guo et al., 2003) and N deficiency is another abiotic stress determining stomatal closure and inhibition of photosynthesis (Lopes and Araus, 2006). An obvious physiological link between N metabolism and control of stomatal movement is provided by the role played by different N sources on the production of Nitric Oxide (NO), a key signal controlling guard cells functioning as well as many other plant development and stress response processes (Wendehenne and Hancock, 2011; Trevisan et al., 2014; Thalineau et al., 2016). NO is a small ubiquitous second messenger required for the ABA-dependent induction of stomatal closure (Desikan et al., 2002; Garcia-Mata and Lamattina, 2002; Bright et al., 2006; Kolla et al., 2007). The synthesis of NO in plant cells remains still a matter of debate and the two plant NO production routes can be classified into the L-arginine dependent pathway (oxidative pathway) and NO_2_^−^ dependent pathway (reductive pathway). However, the reported genetic characterization of *A. thaliana* mutants, indicate Nitrate Reductase1 (NR1) and Nitric Oxide-Associated1 (NOA1) as the main actors affecting NO content in plants (Bright et al., 2006; Lozano-Juste and León, 2010). However, no final proofs have been reported for a NOA1-catalyzed NOS activity and more in general for a NOS activity in plants (Moreau et al., 2008; Jeandroz et al., 2016). AtNOA-1 is a GTPase protein, whose activity is necessary but not sufficient for its function in planta, suggesting that the reduced NO accumulation observed in the *Atnoa1* is due to a defective ribosome/RNA assembly leading to increased production of Reactive Oxygen Species (ROS; Moreau et al., 2008). Nitrate Reductase is a multi-domain, multi-redox enzyme that catalyzes the first reaction of the nitrate assimilation pathway in the cytosol by transferring electrons from NAD(P)H and reducing nitrate to nitrite, which is then transported into the chloroplast where it is further reduced to ammonium. In addition, NR is able to convert nitrite to NO in an NAD(P)H-dependent reaction (Dean and Harper, 1988; Neill et al., 2003; Bright et al., 2006; Crawford, 2006). Many reports point to Nitrate Reductase (NR) as the most important source of NO (Gupta et al., 2011; Mur et al., 2013; Chamizo-Ampudia et al., 2016), although in physiological conditions nitrite is poorly concentrated in contrast to nitrate and alternative putative enzymatic and non-enzymatic routes for NO using NO_2_^−^ as a substrate have been proposed (Bethke et al., 2004; Wang et al., 2010; Santolini et al., 2017).

We recently reported the characterization of *L. japonicus* transgenic PII overexpressing plants that display alterations of the nodular N-fixation activity as well as the N dependent signaling pathway controlling the competence of Lotus plants for nodule formation (D'Apuzzo et al., 2015). In this article, we now investigate whether the un-coupling of PII from its native regulation may affect another process where N assimilation pathways play a crucial role such as the stomatal movement. We report here that overexpression of the PII protein in *L. japonicus* transgenic lines triggers constitutive stomatal closure via induction of the NR/NO biosynthetic pathway leading to phenotypic traits correlated with water conservation and photosynthetic potential. The mechanism of action of the overexpressed PII and its putative physiological role in higher plants is discussed.

## Materials and methods

### Plant material and growth conditions

All experiments were carried out with *Lotus japonicus* ecotype B-129 F9 GIFU. Sterilized seeds were sown on H_2_O agar plates and left over night at 4°C cap side down. After 24 h in the dark in the growth chamber, Petri dishes were exposed to light and kept in a vertical position. Care was taken to maintain the young emerging roots in contact with the filter paper. *In vitro* cultures were maintained in a growth chamber with a light intensity of 200 μmol m^−^^2^ s^−1^ at 23°C with a 16 h/8 h day/night cycle. Solid growth media had the same composition as that of B5 medium (Gamborg, 1970), except that, when needed, (NH_4_)_2_SO_4_ and KNO_3_ were omitted and replaced by ammonium nitrate (NH_4_NO_3_). KCl was added to the medium to replace the potassium source. The media contained vitamins (Duchefa catalog G0415) were buffered with 2.5 mM MES and pH was adjusted to 5.7 with KOH.

For RWC analyses, plants from *in vitro* cultures were transferred to the growth chamber with a 16 h/8 h day/night cycle in pots (2 plant per pot) containing 70 g of a sand/vermiculite mixture (3:1). The plants were watered every 2 days with 30 ml of half strength BandD (Broughton and Dilworth, 1971) solution containing 5 mM NH_4_NO_3_ for three additional weeks until the starting of drought treatment by stopping irrigation.

Plants were grown under greenhouse conditions for Scanalyzer 3D platform in pots of 2 L (16 cm diameter, 19 cm height; one plant per pot), containing 1.5 kg of soil consisting of a 50:50 mixture of peat moss and river sand. Growth conditions were 23/17°C day/night temperature, 75%, relative humidity, and 16 h per day photoperiod. Plants were irrigated with 100 ml of water every 3 days during the analyses.

### Image based phenotyping: data acquisition and processing

Phenotyping through image analysis was performed with a LemnaTec Scananalyzer 3D System. Images of Lotus plant chlorophyll fluorescence were taken under illumination of light having wavelengths greater than 500 nm and with a filter in front of the camera to remove wavelengths shorter than 500 nm. The images of fluorescent light from the Lotus plants were then converted from the red/green/blue (RGB) color space to the hue/saturation/value color space. The color pixels of the plants were divided into a scale of 13 classes. When a plant is placed under greater levels stress more fluorescent light of higher energy (or shorter wavelength) is released and this change might be measured through pixel distribution. To quantify this shift a photosynthetic health index is calculated with the formula (F_x_ − *F*_y_)/(F_x_ + *F*_y_), where F is the number of pixels in a specific color class while x and y are the color classes. The color classes chosen were determined experimentally for each experiment by examining the hue histogram.

Bio-volume parameters were measured as described by Eberius and Lima-Guerra (2009).

### Plant transformation

We followed the procedures previously described (Lombari et al., 2003; Barbulova et al., 2005).

### T-DNA constructs preparation

Oligonucleotides and strategies for pr*LjGLB1*-*gus*A, *LjGLB1*-GFP fusions and PII over-expressing T-DNA constructs were described in D'Apuzzo et al. (2015).

### Confocal analysis

Confocal microscope analyses were performed using a Nikon PCM2000 (Bio-Rad, Germany) laser scanning confocal imaging system. For GFP and RFP detection, excitation was at 488 nm and detection between 515 and 530 nm. For the chlorophyll detection, excitation was at 488 nm and detection over 570 nm.

### Quantitative real-time RT-PCR

Total RNA was prepared from Lotus leaves using the procedure of Kistner and Matamoros (2005). The samples were treated with DNAse I (Qiagen) to remove contaminating DNA the absence of which was subsequently confirmed by PCR. One microgram of total RNA was annealed to random decamers and reverse-transcribed with reverse transcriptase (Qiagen) to obtain cDNA. Real time PCR was performed as described in Omrane et al. (2009) with a DNA Engine Opticon 2 System, MJ Research (MA, USA) using SYBR to monitor dsDNA synthesis. The ubiquitin (*UBI*) gene (AW719589) was used as an internal standard. The concentration of primers was optimized for each PCR reaction and each amplification was carried out in triplicate. The PCR program used was as follows: 95°C for 3 min and 39 cycles of 94°C for 15 s, 60°C for 15 s, and 72°C for 15 s. Data were analyzed using Opticon Monitor Analysis Software Version 2.01 (MJ Research). The qRT-PCR data were analyzed using comparative Ct method. The relative level of expression was calculated with the following formula: relative expression ratio of the gene of interest is 2^−ΔCT^ with ΔCT = Ct_AMT1_ minus CT_UBI_. The efficiency of the different AMT1 primers was assumed to be 2.

Analysis of the melting curve of PCR product at the end of the PCR run revealed a single narrow peak for each amplification product, and fragments amplified from total cDNA were gel-purified and sequenced to assure accuracy and specificity. The oligonucleotides used for the qRT-PCR are the following: LjNR-forw 5′-TGCAGAACTTGCCAATGAAG-3′; LjNR-rev 5′-GTTTCTCCACCGTCCAGTGT-3′.

### Histochemical GUS analysis

Histochemical staining of whole plant material was performed as described by Rogato et al. (2010) and Ferraioli et al. (2004).

### Stomatal aperture bioassays

To follow ABA induced stomatal closure, epidermal strips were prepared from well-watered 3- to 4-week-old plants at the 6th hour of the daily light period. Strips are incubated in 30 mM KCl, 10 mM MES-KOH, pH 6.5, at 22°C, and exposed to light (300 μmol m^−2^ s^−1^) for 3 h in the presence or in the absence of 10 μM ABA. Stomatal apertures were measured using a Nikon Optiphot-2 microscope (http://www.nikon.com/) fitted with a digital camera and a TG 1017 digitizing table (Houston Instruments; http://www.tms-plotters.com) linked to a personal computer. To study ABA induced inhibition of stomatal opening, leaves were harvested in darkness at the end of the night period, epidermal strips prepared and stomatal measurements performed as above with/out 10 μM ABA. To measure constitutive stomata aperture, homogeneous stem cuttings were obtained from well-watered 3- to 4-weeks-old plants at the 6th hour of the daily light period. The stem bases were immediately sealed with instant adhesive glue on a microscope cover glass and leaves were detached at T0, 1, and 2 h after sealing for epidermal strips preparation. For each treatment, at least 60 stomatal apertures were measured and data presented are the mean of at least 3 independent experiments (2 leaves per experiment).

### Water loss determination

Homogenous stem cuttings from 3 to 4 weeks old wild type and transgenic plants, carrying 4–5 trifoliates from the top second or top third internodes were detached. The stem bases were immediately weighted and sealed with instant adhesive glue on a microscope cover glass. Dehydration and weight measurements at the designated time intervals were performed in a dark room. Water loss is described as the percentage of initial fresh weight. Five replicates from different individual plants were analyzed.

### Relative water content (RWC) determination

To measure RWC, 24 leaves per replicate were used. RWC was calculated using the formula: RWC (%) = ((FW − DW)/(TW − DW)) × 100 (Hewlett and Kramer, 1962). Leaves of corresponding trifoils were detached from wild type and transgenic plants and immediately weighed to determine their FW (fresh weight). The same leaves were weighted after 24 h floating in deionized water and blotted dry to determine their TW (turgid weight). DW (dry weight) measurements were taken after the leaves were oven-dried (65°C) in paper bags for 24 h. Each biological replicate (four) consisted of a pool of leaves from two plants that were grown in the same pot.

### Fluorometric NO quantification in Lotus guard cells

The cell-permeable diacetate derivative diamino-fluorescein-FM (DAF-FM DA) (Sigma-Aldrich Cod. D2321) was used as a specific fluorescent probe for the detection of intracellular NO (Gould et al., 2003). Epidermal strips obtained from leaves of adult Lotus plants grown in pots were prepared accordingly to Behera and Kudla (2013) incubated for 10 min in the loading buffer (LB) (30 mM KCl, 10 mM MES-KOH). The buffer was then replaced with fresh LB supplemented with 5 μM DAF-FM-DA (0.1% v/v final DMSO) and kept for 15 min in the dark. The epidermal strips were then washed twice (2 × 5 min) with fresh LB before being visualized by microscopy.

For NO detection analysis, Lotus guard cells loaded with DAF-FM-DA were imaged by an inverted fluorescence microscope Nikon Ti-E (Nikon, JP, http://www.nikon.com/) with a 20 × A.N.0, 75 Plan Apo VC dry objective. Excitation light was produced by a fluorescent lamp Prior Lumen 200 PRO (Prior Scientific, UK, http://www.prior.com). The specimens were excited at 470/40 nm and the DAF-FM-DA fluorescence was collected with a bandpass filter of 505–530 nm. The fluorescence emitted was collected with an Hamamatsu Dual CCD Camera ORCA-D2 (Hamamatsu, Photonics, JP, http://www.hamamatsu.com/). Exposure time was 200 ms with a 1 × 1 CCD binning. The NIS-Element (Nikon, JP, http://www.nis-elements.com/) was used as platform to control microscope, illuminator, camera and post-acquisition analyses. Pixel intensity analysis of guard cells loaded with DAF-FM-DA was determined with FIJI (https://fiji.sc/). The DAF-FM-DA fluorescent signal was background subtracted.

### Nitrate reductase assay

Leaf extracts of wild type and PII over-expressing plants were prepared in 100 mM Tris-HCl pH 8.0, 10 μM FAD, 1 mM EDTA, 1 mM dithiothreitol (DTT), 1 mM phenylmethylsulphonylfluoride (PMSF) and 0,1% Triton X-100. The extracts were assayed for Nitrate Reductase activity as described in Pajuelo et al. (2002).

### Statistical analysis

Statistical analyses were performed using the VassarStats analysis of variance program.

## Results

### *LjGLB1* is strongly expressed in guard cells

**Figure 1:**
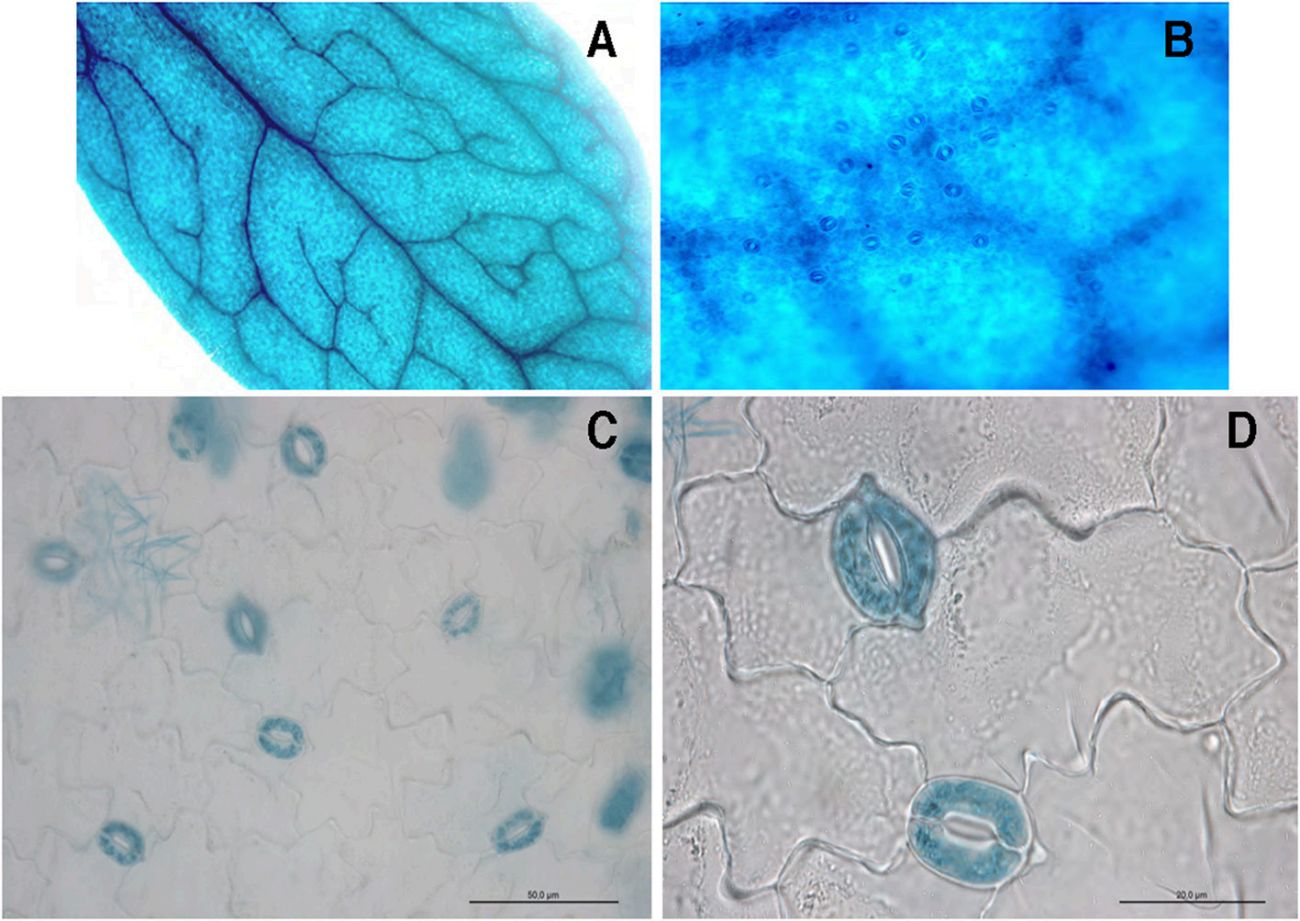
Optical microscopy of a representative leaf of *L. japonicus* transgenic plant transformed with the pr*LjGLB1-gus*A construct. **(A)** Whole mount leaf showing staining in central and lateral veins and mesophyll cells. **(B)** Higher magnitude of leaf in **(A)** showing stained guard cells. **(C)** Peeled epidermis strip after staining. **(D)** Higher magnitude of epidermis strip in **(C)**.

We have recently described the GUS activity driven by the pr*LjGLB1-gus*A promoter-fusion, localized in vascular bundle of *Lotus japonicus* transgenic hairy roots and nodules with a slight activity observed in root tips (D'Apuzzo et al., 2015). In order to gain further inside the profile of *LjGLB1* expression in different organs, we have used the same T-DNA construct to obtain *L. japonicus* transgenic plants via *A. tumefaciens*-mediated transformation. Three different Lotus transgenic lines have been obtained after transformation with the pr*GLB1*-*gus*A fusion containing the 980 bp fragment upstream of *LjGLB1* ATG start codon. These three transgenic lines have been propagated to isolate homozigous T2 lines with consistent patterns of GUS activity. The analysis of the *pLjGLB1-gus*A confirms the expression previously described in vascular bundle of transgenic roots (D'Apuzzo et al., 2015), giving the opportunity to identify an additional striking spatial profile of expression in leaf tissues where GUS activity is localized in main and secondary veins but also in mesophyll and guard cells (Figures 1A,B). The GUS staining in guard cells is even better visualized in leaves after epidermis peeling (Figures 1C,D). The analysis of the 980 bp at the 5′ region of the *LjGLB1* gene reveals some interesting features consistent with this spatial profile of GUS activity. This region contains eight (A/T) AAAG and three CCAAT elements, binding motifs for Dof zinc finger and NF-WA transcriptional factors (Figure S1) that have been associated to guard cell activity in potato, grapevine and *A. thaliana* (Li et al., 2008; Galbiati et al., 2011).

**Figure 2:**
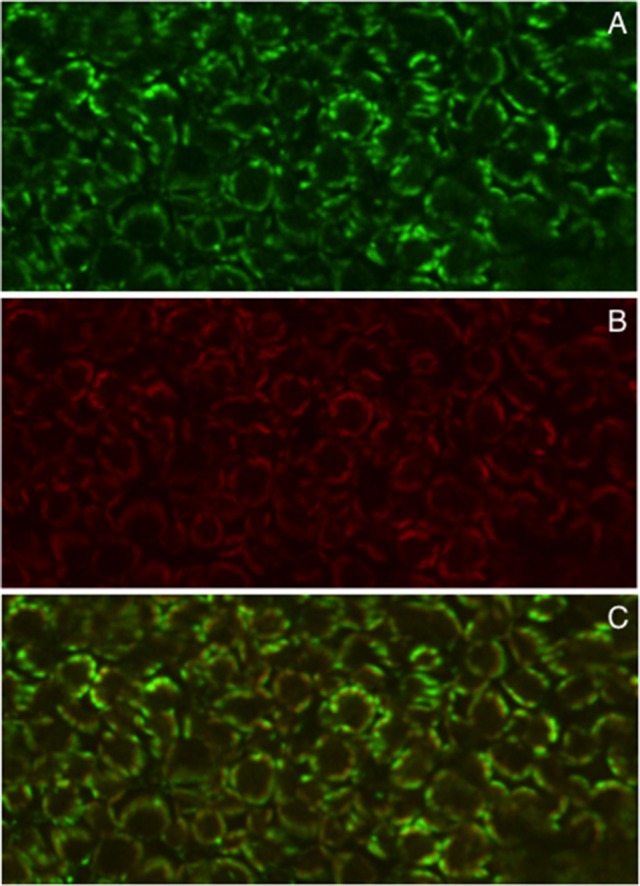
Chloroplast localization of 35S-GLB1-GFP at the confocal laser scanning. **(A)** Green fluorescence. **(B)** Red fluorescence for chlorophyll localization. **(C)** Co-localization by merging images.

*A. tumefaciens*-mediated transformation-regeneration procedure has been also exploited to obtain Lotus trangenic plants transformed with *LjGLB1-GFP* fusion driven by the CAMV35S constitutive promoter and confocal laser-scanning fluorescence analysis in T2 transgenic lines indicated unambiguously a chloroplast localization of GFP in leaves, which co-localizes with chlorophyll red autofluorescence (Figure 2), confirming previous data obtained in Arabidopsis and rice (Hsieh et al., 1998; Sugiyama et al., 2004).

### PII overexpression affects stomatal movement

**Figure 3:**
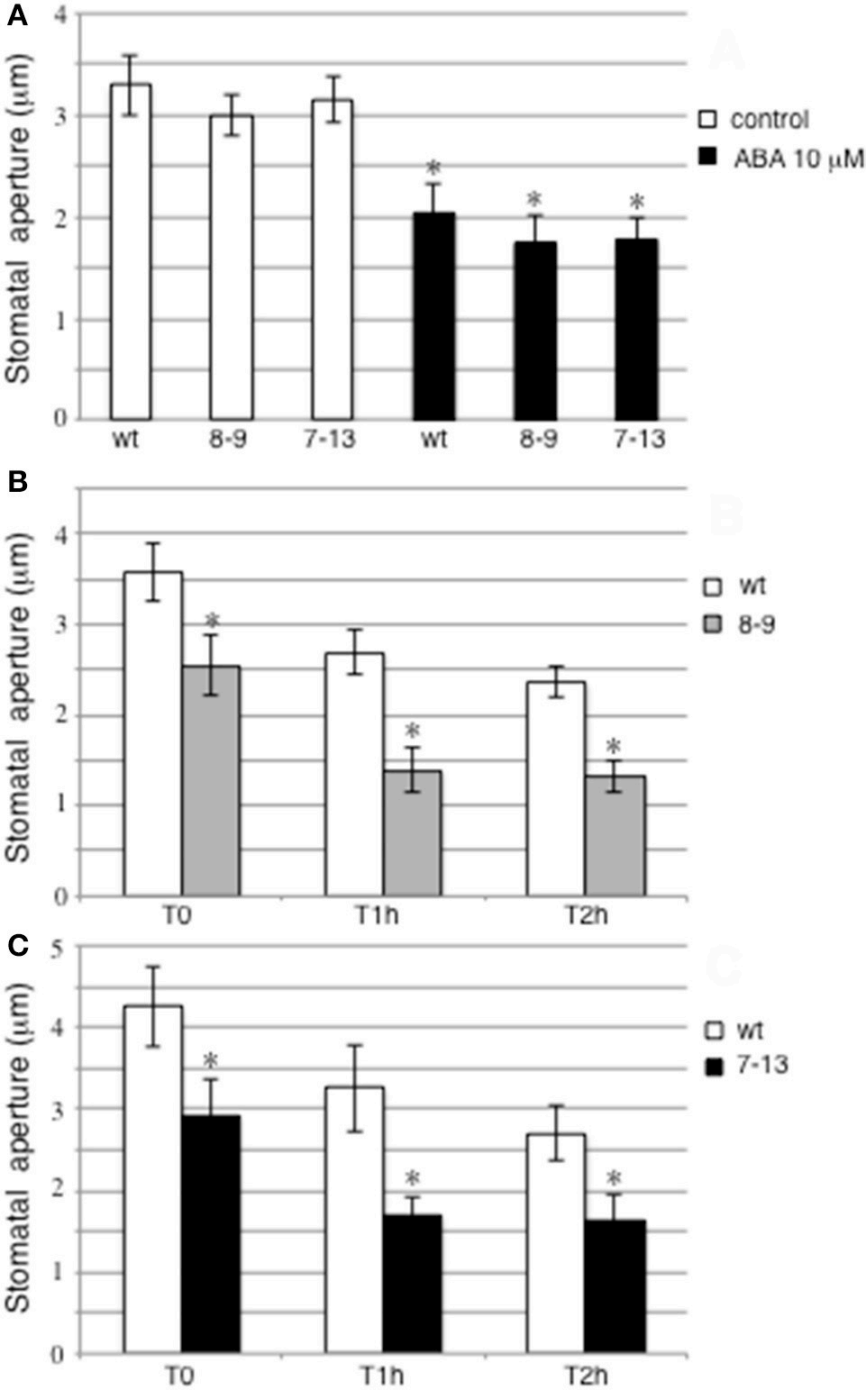
Analysis of stomata movement in wild type and PII over-expressing transgenic lines. **(A)** Effect of 10 μM ABA on stomata closure. Epidermal strips from detached leaves were exposed to light for 3 h and then treated with (black bars) and without ABA (white bars) for an additional hour. Asterisks indicate significant differences between ABA-treated and un-treated plants (*p* < 0.0001; VassarStats analysis of variance program). **(B,C)** Stomata aperture in detached leaves from stems cutting isolated at the 6th hour of the daily light period and sealed at the bases to avoid water loss by gravity at T0, 1, and 2 h after cutting. Wild type and transgenic lines are indicated. Asterisks indicate significant differences between transgenic and wild type plants (*p* < 0.0001; VassarStats analysis of variance program). Bars represent the average and SE for three independent experiments. For each treatment, at least 60 stomatal apertures were measured and data presented are the means of at least 3 independent experiments (2 leaves per experiment).

The clear-cut pattern of pr*LjGLB1-gusA* activity detected in guard cells prompted us to investigate whether PII could play any role in the control of stomata movement. Therefore, we took advantage of the genetic tool we recently characterized, represented by two transgenic lines ectopically expressing *LjGLB1* under the control of the CAMV35S constitutive promoter (D'Apuzzo et al., 2015), to test whether the PII overexpression could affect the guard cell functioning. Epidermal fragments have been prepared from leaves of wild type plants and the two PII-overexpressing 7–13 and 8–9 transgenic lines and analyzed for stomatal movement. We have first analyzed the effect of 10 μM abscisic acid (ABA) on stomatal closure phenotype in epidermal strips of detached wild type and PII-overexpressing transgenic leaves incubated for 3 h on light (300 μmol m^−^^2^ s^−^^1^) with or without addition of 10 μM abscisic acid (ABA). These measures indicate, as expected, an ABA dependent triggering of stomatal closure (40%) but no statistically significant differences are revealed in guard cells aperture of detached wild type and PII over-expressing leaves (Figure 3A). Therefore, PII overexpression is not affecting the level of the ABA-induced stomata closure in Lotus plants. Through a different approach, we have then tested the effect of PII overexpression on stomata closure in a time course experiment during a rapid leaf dehydration treatment. Stem cuttings 6–7 cm long, from 4 to 5 weeks old wild type and transgenic plants, carrying 4–5 trifoils, have been isolated at the 6th hour of the daily light period and immediately sealed at the wounded site with instant adhesive glue. Central leaves from corresponding trifoils detached at T0, 1, and 2 h have been utilized for epidermal strips preparation and immediate stomatal measurement. Data shown in Figures 3B,C indicate a progressive stomatal closure triggered by this rapid dehydration treatment in wild type and transgenic leaves but, a clear-cut increase of stomatal closure is observed in 8–9 and 7–13 transgenic leaves compared to wild type leaves and this effect is displayed from the T0 time point (71 and 68% of wild type stomatal aperture, respectively). The leaves for stomata measurement at T0 are harvested immediately after the stem cutting and sealing (less than 3 min), indicating that the detected differences in stomata apertures are likely associated to a constitutive condition of PII overexpressing leaves rather than to a differential early response to drought treatment. It should be also considered that the unchanged measures of stomatal aperture in the detached leaves of control plants not treated with ABA (white bars in Figure 3A) are due, in that experimental set up, to the preliminary treatment of epidermal strips that are exposed 3 h to direct light, inducing full aperture and hence equalizing the stomatal starting conditions.

*L. japonicus* lines with LORE1 retrotransposon element inserted within the *LjGLB1* sequence are not available in the large collection of insertion lines recently released (Fukai et al., 2012; Urbanski et al., 2012; Malolepszy et al., 2016). Therefore, in order to test the possible physiological involvemen of PII protein in the guard cell functioning we have analyzed the stomatal aperture phenotype of an *Arabidopsis thaliana* knock out mutant already characterized for different phenotypes (Ferrario-Mèry et al., 2005). The T-DNA insertion line N521878 (Salk collection) carrying a double insertion event in the fourth intron of the *AtGLB1* gene and previously characterized with an undetectable expression of the *AtGLB1* gene (PIIS2 mutant; Forchhammer and Hedler, 1997), has been obtained from the Nottingham Arabidopsis Stock Center (http://arabidopsis.info). We have confirmed by PCR the genotype of homozygous insertion mutants and tested the stomata phenotype by comparison with leaves of wild type *A. thaliana* Columbia ecotype. This analysis did not reveal any alteration neither of ABA-induced nor constitutive stomata aperture in the PIIS2 plants (Figure S2).

### PII overexpression induces constitutive changes on transpirational water loss and photosynthetic performances

Stomata serve as major gateways both for transpirational water loss and CO_2_ influx of plant leaves. Therefore, we have investigated whether the altered stomatal movement observed in Lotus PII overexpressing plants could affect two phenotypes correlated to those physiological functions such as water use efficiency and photosynthetic performances.

**Figure 4:**
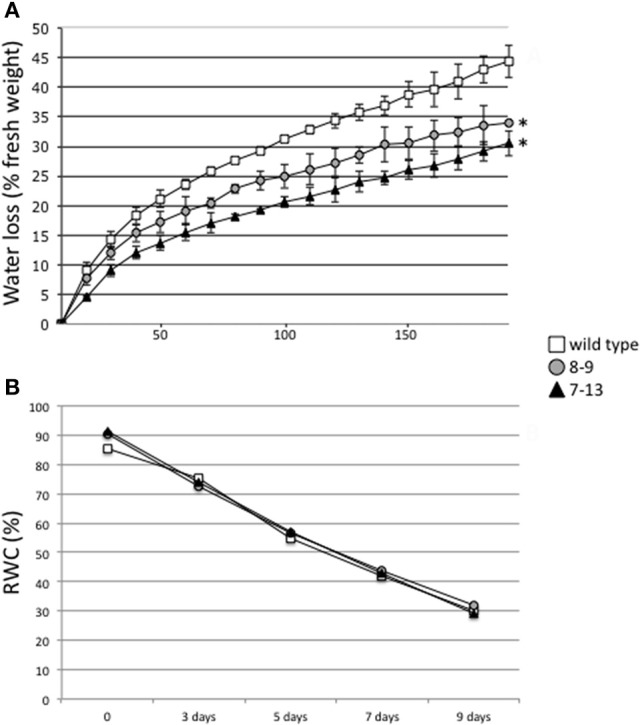
Hydric stress responses in wild type and PII over-expressing transgenic lines. **(A)** Time course of water loss from stem cuttings of wild type, 7–13 and 8–9 lines. Water loss is expressed as the percentage of initial fresh weight. Values represent means and standard errors of 5 experiments with plant samples of different plants. Asterisks indicate significant differences between transgenic and wild type plants (*p* < 0.001; VassarStats analysis of variance program). **(B)** Changes in RWC of wild type, 7–13 and 8–9 lines during drought. Each value represents the mean of 20 measurements with standard errors. Plant genotypes are indicated: wild type (square), 8–9 line (circle), 7–13 line (triangle).

In order to test whether the constitutive reduced aperture of stomata in the PII overexpressing leaves induce a favorable response to a rapid dehydration treatment, the stem cuttings material described above, has been used for the analysis of water loss. The bases of stem cuttings are immediately sealed with instant adhesive glue on a microscope cover glass to prevent water loss by gravity and fresh weight measured in the dark, throughout the desiccation treatment. As shown in Figure 4A, both 7–13 and 8–9 transgenic lines display an early significant reduced transpirational water loss in response to this rapid drought stress treatment. In the following analysis, we have tested whether wild type and transgenic PII overexpressing plants display differences in the gradual long term response to cessation of irrigation. Plants have been grown individually in the growth chamber, in pots with soil composed by a mixture of sand/vermiculite and watered with half strength B&D medium supplemented with 5 mM NH_4_NO_3_. The plants in vegetative growth conditions, either wild type, –9 and 7–13, after 12 days of desiccation showed severe wilting and turned brown with no differences observed in the recovery capacities during re-watering treatments (data not shown). This observation has been quantified through the analysis of leaf Relative Water Content (RWC) that progressively decrease with overlapping curves, in both wt and transgenic leaves during drought stress treatment (Figure 4B).

**Figure 5:**
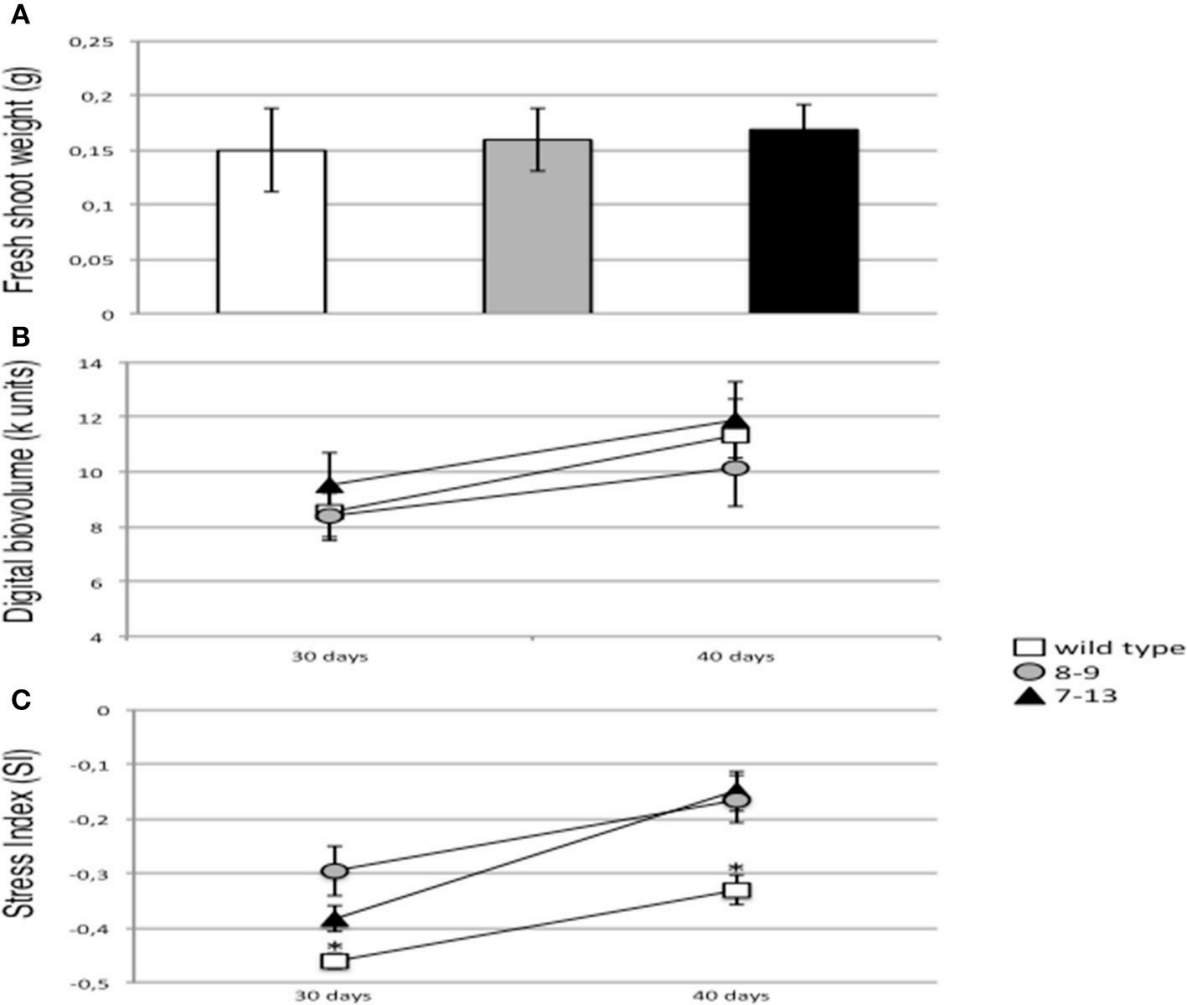
Biomass parameters and stress index in normal watering vegetative growth conditions. **(A)** Shoot fresh weights of wild type (white bar), 8–9 (gray bars) and 7–13 (black bars) plants under growth chamber conditions. Data represent the mean and standard errors obtained from 3 independent experiments (10 plants per experiment). **(B)** Digital bio-volumes of wild type (white square), 8–9 (gray circle) and 7–13 (black triangle) plants under greenhouse conditions. The measures refer to the sum of the projected areas (in pixels) of the plant leaves from the two orthogonal side views and the natural logarithm of one third of the projected plant area from the top view. Data points represent the mean and standard errors obtained from three plant replicates. **(C)** Stress index of wild type (white square), 8–9 (gray circle) and 7–13 (black triangle) plants under greenhouse conditions. Data points represent the mean and standard errors obtained from six images (two images per plant). Florescence re-emission has been measured with a Scanalyzer 3D platform (LemnaTec). Asterisk indicates significant differences between wild type and transgenic plants (*p* < 0.0001; VassarStats analysis of variance program).

The detected reduced stomata aperture of PII overexpressing plants is not associated to the display of macroscopic morphological changes when shoot growth parameters are compared in wild type and transgenic plants grown in axenic conditions (D'Apuzzo et al., 2015). We have confirmed this result through the analyses conducted on the normally watered control plants maintained in growth chamber conditions and used for the RWC analysis, as shoot fresh weight doesn't change between wild type and transgenic plants (Figure 5A). This phenotype has been even further investigated through a quantitative analysis of bio-volume parameters performed with a Scanalyzer 3D platform on wild type and transgenic plants, grown in greenhouse conditions and normally watered. Groups of 12 wild type, 8–9 and 7–13 plants have been transferred to pots and grown vegetatively for 50 days under greenhouse conditions. Leaf bio-volume parameters are obtained in a non-destructive way by calculating the sum of the projected areas (pixels) from the two orthogonal side views, during a time span of 10 days (from day 30 to day 40). The values plotted on Figure 5B confirm no significant differences between wild type and transgenic PII overexpressing plants. The plants grown under greenhouse conditions have been further analyzed by taking specific measures of their photosynthetic potential. Under visible light illumination, leaf pigments of the photosystems adsorb the energy of the photons in their electrons by elevating them to higher orbitals. There are three competing fates for this energy: (i) phytochemistry, the productive phase of photosynthesis, (ii) dissipated as kinetic energy, heat, or (iii) florescence, re-emitted as luminous energy at lower energy (Oxborough, 2004). The interplay of these three pathways for the photon energy depends on the state of the plant and normal healthy plants will be consuming the energy in photosynthesis, whereas stressed plants will be dumping the energy as heat or fluorescence. Therefore, leaves fluorescence is a quantitative parameter of their photosynthetic potential and these measures might be utilized for evaluation of the state of plant stress index (Muller et al., 2001). The values of the photosynthetic stress index plotted on Figure 5C and measured at the same time points used for the bio-volume analyses, should be considered only as relative levels when compared to other plants in the same experiment. The bins for the stress index calculation are chosen around the peak of the Hue histogram such that the calculated values, ranging from −1 to +1, indicate whether the median peak has shifted to the left (red shifted, toward −1) or to the right (blue shifted, toward +1) with respect to the wild type control. In this manner plants that are more stressed have higher values with respect to the reference plants. Interestingly, the measures fall all in a positive range of the scale values, confirming the absence of evident stressful conditions neither in wild type nor transgenic plants but nevertheless, both transgenic PII overexpressing lines show a significant higher stress index when compared to wild type plants (Figure 5C).

### Physiological bases of the stomata movement alteration induced by PII overexpression

**Figure 6:**
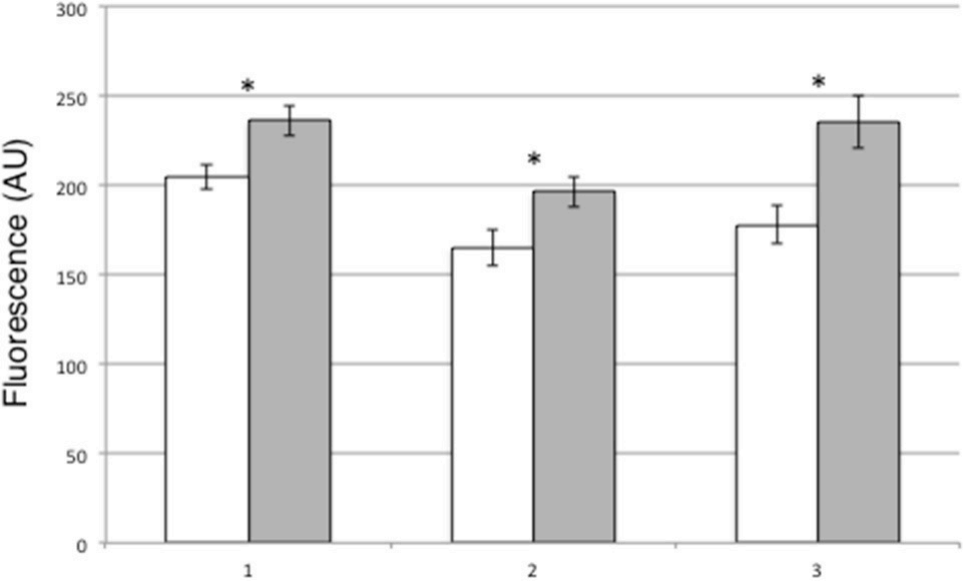
NO production in DAF-FM loaded leaves of wild type and 8–9 leaves. DAF-FM-DA fluorescence is indicated as pixel intensity arbitrary units (AU). Wild type (white bars), 8–9 over-expressing lines (gray bars). (1–3) Bars represent the average and SE for three independent experiments (*n* > 85 guard cells per experiment and genotype). Asterisks indicate significant differences (1, *p* < 0.002; 2, *p* < 0.005; 3, *p* < 0.0001; VassarStats analysis of variance program).

A possible role of PII in guard cells functioning is particularly intriguing as this protein has been already involved in *A. thaliana* on the control of NO_2_^−^ and arginine content, two main sources of NO synthesis in leaves (Ferrario-Mèry et al., 2005, 2008). Therefore, we have tested whether the content of nitric oxide (NO), a key signal controlling the stomatal closure, is affected in leaves of trangenic overexpressing PII plants compared to wild type. Intracellular NO production has been compared in wild type and 8–9 transgenic leaves by means of the cell-permeable DAF-FM DA fluorescent probe. Peeled leaf epidermal fragments have been obtained from Lotus plants grown in the growth chamber and loaded with the dye. The excess of DAF-FM-DA probe is washed out and the epidermal strips are immediately analyzed by means of wide field fluorescence microscopy. As shown in Figure 6, guard cells of 8–9 leaves have a significantly increase of NO accumulation when compared to wild type cells (about 20%), thus indicating a striking correlation between NO generation and stomatal closure observed in the PII overexpressing transgenic plants.

**Figure 7:**
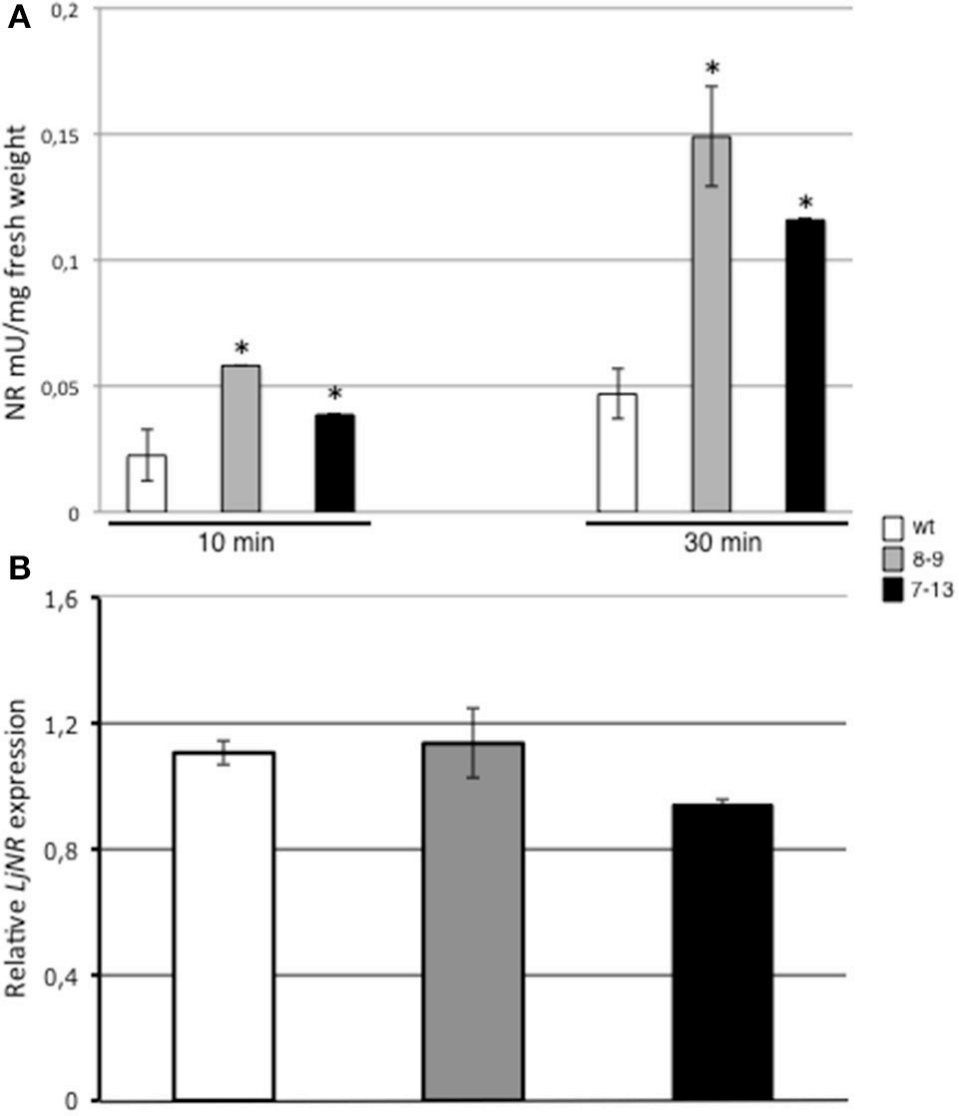
NR enzyme activity and transcript levels in leaves of *L. japonicus* wild type and PII over-expressing plants. **(A)** NR activity in leaves of 3 weeks old plants grown *in vitro* on B5/2 Gamborg medium. Spectrophotometric values were measured at 10 and 30 min from the starting of reaction in wild type, 7–13 and 8–9 lines. Data bars represent means and standard deviations of 4 samples from different plants. Asterisks indicate significant differences between wild type and transgenic plants (*p* < 0.0001; VassarStats analysis of variance program). **(B)** Semi-quantitative RT-PCR analysis of *LjNAR* expression. Total RNAs were prepared from leaves of the same plants tested for NR activity. *L. japonicus* encoded UBI was used as a reference gene to normalize the expression of *LjNAR*. Plant genotypes are indicated. Data bars represent means and SE of the values obtained from 4 biological samples.

In order to further characterize the altered biosynthetic pathways leading to the constitutive increased NO content in leaves of *L. japonicus* PII over-expressing plants (Figure 6), we have focused our attention on nitrate reductase (NR), a crucial enzyme for production of NO_2_, an endogenous substrate for NO synthesis in leaf tissues. Nitrate reductase activities have been assayed in leaves of 3 weeks old Lotus plants grown in axenic cultures in the presence of 12 mM KNO_3_ and 0.5 mM (NH_4_)_2_SO_4_ as N sources (B5/2 Ganborg medium). NR activity is significantly increased in both 7–13 and 8–9 PII over-expressing lines when compared to wild type plants (Figure 7A; 63 and 70% increased activity, respectively). In a parallel analysis, we have also tested in the same plants used for characterizing the enzymatic activity, the *LjNR* transcript abundance. The *L. japonicus* gene coding for NR has been previously identified and characterized at the molecular level (Kato et al., 2003) and the blast analysis against the new version 3.0 of the *Lotus japonicus* genome database assembly (http://www.kazusa.or.jp/lotus) confirms the Lj0g3v0006719.1 as the unique *LjNR* gene. Data reported on Figure 7B show no significant differences of *LjNR* expression between wild type and PII over-expressing plants, indicating that overexpressed PII protein affects only NR enzymatic activity. The same analyses have been also conducted on leaves of plants grown in soil under growth chamber conditions with identical results (data not shown).

## Discussion

The striking expression of *LjGLB1* in guard cells revealed by the analysis of the spatial profile of its promoter activity (Figure 1) suggests a physiological role of the PII protein in that cellular context. A significant expression of the *Arabidopsis thaliana AtGLB1* gene (At4g01900) in guard cells has been also previously reported by array analysis but that observation was not further developed (Leonhardt et al., 2004).

Our results indicate that the PII overexpression in Lotus plants is triggering an increase of NR activity in leaves of the two transgenic lines (Figure 7A), which can be considered causative of the significant induction of the leaf NO content observed in leaves of the 8–9 line when compared to wild type plants (Figure 6). In higher plants PII has been already involved in the control of the arginine biosynthetic pathway, through interaction with NAGK (Sugiyama et al., 2004; Chen et al., 2006; Ferrario-Mèry et al., 2006) and such a role could also imply a cross-talk with the NO production biosynthetic routes. Consistently, we have reported that the two PII overexpressing Lotus transgenic lines have a positive effect on the ornithine/arginine biosynthetic pathway displaying an increased citrulline content that could be also correlated to the NO accumulation reported in this work (D'Apuzzo et al., 2015).

These results arise the question of how the alteration of the *LjGLB1* regulatory profile of expression in *L. japonicus* 8–9 and 7–13 transgenic lines might be responsible of the increased content of NO via induction of NR activity. Different hypotheses must be taken into account to explain the effects of PII overexpression. Inhibition of NR by post-translational modification (through phosphorylation) varies in parallel with NO production in plants, indicating that the crucial participation of NR in NO production, is mainly controlled by the activation state of the NR enzyme. Therefore, one possibility could be that NR represents a not yet identified PII target in plants, whose enzymatic activity can be controlled through a post-translational regulation via direct PII/NR protein/protein interaction. However, this hypothesis seems inconsistent with some biochemical and genetic characterizations conducted in *A. thaliana*. Affinity chromatography and mass spectrometry experiments carried out in *A. thaliana* did not identify the NR enzyme as a PII interacting protein (Chen et al., 2006) and *A. thaliana* PII knock out mutants don't display an altered NR activity when compared to wild type plants (Ferrario-Mèry et al., 2005). Nevertheless, general conclusions based on phenotypic characterizations of *A. thaliana* PII mutants could be biased by the observation that Arabidopsis and *Brassicaceae* plants don't identify the true prototype of the plant PII protein (Chellamuthu et al., 2014). An alternative model that fits the phenotypes displayed by Lotus PII overexpressing plants and Arabidopsis mutants (Ashraf and Harris, 2013) is based on an indirect action of PII on NR. Nitrite transport into the chloroplasts as well as its metabolic conversion, is a highly efficient and finely tuned process to avoid its accumulation in the cytosol, allowing optimal utilization of nitrate. *A. thaliana* PII mutants have been reported as more sensitive to nitrite toxicity (Ferrario-Mèry et al., 2005) and this phenotype is associated to an increased incorporation of ammonium and amino acids in isolated chloroplasts independent by changes on Glutamine Synthetase (GS)/Glutamine Oxoglutarate Aminotransferase (GOGAT) activities, suggesting a negative role of PII on the control of nitrite uptake into chloroplasts of higher plants (Ferrario-Mèry et al., 2005). NR-induced NO production is thought to become crucial only in specific conditions in which NO_2_^−^ accumulates (Yamasaki and Sakihama, 2000; Santolini et al., 2017) and PII overexpression could determine a reduced uptake of NO_2_^−^ into the chloroplasts with a consequent induction of NR activity due to increase of the NO_2_^−^ cytosolic content (Kaplan et al., 1978; Rockel et al., 2002). Interestingly, transgenic tobacco plants expressing the antisense of the Nitrite Reductase gene (NiR) accumulate 10-fold higher nitrite concentrations with a 100-fold higher NO emissions than in wild type plants (Morot-Gaudry-Talarmain et al., 2002), thus sharing a similar phenotype with Lotus PII overexpressing plants.

As a matter of fact, the PII overexpression in our Lotus transgenic plants, induces an increased stomatal closure that is not observed in the *A. thaliana* PIIS2 null mutant (Figure S2). Our results indicate a constitutive effect on stomatal closure in leaf epidermal strips of transgenic plants when compared to wild type (Figures 3B,C) and these differences are maintained for at least 2 h during the dehydration treatment conducted on stem cuttings. Conversely, no changes are observed in the pattern of stomatal closure of detached leaves treated with 10 μM ABA (Figure 3A). These results might be explained, considering that the ABA-dependent triggering of stomatal closure occurring through the induction of NO generation via NOA1- and/or NR1-dependent pathways is fully functional in both wild type and PII overexpressing leaves and therefore no changes are expected on stomatal movement in detached leaves treated with 10 μM ABA. On the other hand, the increased stomatal closure is observed in normal growth conditions during the daily light period, when the ABA-dependent pathway should be not switched on and hence the effect mediated by PII overexpression via NR activity can be displayed (Figure 3B).

It is generally acknowledged that reduction of CO_2_ diffusion from the atmosphere to the site of carboxylation due to stomatal closure and reduced mesophyll conductance, contributes to a decrease in photosynthesis (Lawlor and Cornic, 2002; Chaves and Oliveira, 2004; Ashraf and Harris, 2013). We have measured the amount of energy released by plant canopies through fluorescence, which is a parameter indicative of the plant photosynthetic potential as more energy is released when the photosynthesis apparatus is under abiotic stress (Muller et al., 2001). The data reported in Figure 5C show a significant reduction of the mutants photosynthetic potential when compared to wild type and this effect is likely associated to the increased stomatal closure. However, the values plotted in Figure 5C are indicative of a slight stressful condition and this could explain why we don't observe clear-cut deficient shoot biomass phenotypes in the overexpressing plants (Figures 5A,B).

Despite the evidences based on genetic characterization of different plant mutants as well as the employing of NO donors and/or scavengers which strongly support the role played by NO generated in response to ABA for triggering the stomatal closure, the exact contribution of this biosynthetic pathway with regard to water-stress tolerance is still controversial and consistently, neither the Arabidopsis *nia1* nor the *noA1* mutants show an obvious wilty phenotype (Desikan et al., 2002). Drought responses involve physiological and biochemical processes such as changes in gene expression and enzyme activities. Nitrate reductase transcription and enzymatic activity were reported to be reduced in different plants under drought conditions (Ferrario-Méry et al., 1998; Foyer et al., 1998; Correia et al., 2005) and these decreases have been correlated to stomatal closure and consequent lower internal CO_2_ concentration (Kaiser and Fòrster, 1989). Therefore, while NO seems to play a crucial role in the control of guard cells movement in well-hydrated tissues, most likely in the stomatal closure occurring during the light/dark transition; it seems not to be required for the closure of stomata during dehydration. In fact, NO doesn't enhance the drought response in intact Arabidopsis plants, which most likely respond to an ABA dependent signaling pathway that bypasses the NR-mediated NO synthesis (She et al., 2004; Zhang et al., 2007; Neill et al., 2008; Ribeiro et al., 2009). Consistently, our analysis shows that the drought response phenotype, analyzed by estimation of RWC on detached leaves of intact *L. japonicus* plants exposed for several days to interruption of watering is not changed between wild type and transgenic plants (Figure 4B). On the other hand, the water loss analysis indicates that the constitutively increased stomatal closure of the PII overexpressing plants induces an improved immediate response to rapid dehydration treatment (Figure 4A). This result is also consistent with several observations reporting that the prior exposure of plants to NO is ameliorative for the subsequent exposure to drought (Garcia-Mata and Lamattina, 2001; Tian and Lei, 2006; Zhang et al., 2007).

Although overexpression of wild type genes represents a powerful genetic tool for unraveling intricate mechanisms by revealing phenotypes of interest, the interpretation of results must be treated with caution. Therefore, a further characterization of *L. japonicus* PII overexpressing plants will be focused on the obtainment of *L. japonicus* loss of function mutants as a crucial tool for the understanding the physiological involvement of PII in the regulation of the NR-mediated NO biosynthetic route as well as other NR-dependent pathways in different conditions of growth.

## Author contributions

All the authors critically revised the article. APa, VV, LA, ED, APe, and SS performed research and analyzed data; AC, FC, AV, and MC designed research; MC wrote the paper.

### Conflict of interest statement

The authors declare that the research was conducted in the absence of any commercial or financial relationships that could be construed as a potential conflict of interest.

## Supplementary material

Figure S1 Sequence of the 980 bp 5′ UTR region of the *LjGLB1* gene. Putative biding sites for Dof and NF-WA transcriptional factors are indicated in bold and italic, respectively.

Figure S2 Analysis of stomata aperture in *A. thaliana* Columbia ecotype and PIIS2 plants. **(A)** Effect of 10 μM ABA on stomata closure. Epidermal strips from detached leaves were exposed to light for 3 h and then treated with (black bars) and without ABA (white bars) for an additional hour. Asterisks indicate significant differences between ABA-treated and un-treated plants (*p* < 0.0001; VassarStats analysis of variance program). **(B)** Stomata aperture in detached leaves isolated at the 6th hour of the daily light period. Columbia wild type and PIIS2 plants are indicated. Bars represent the average and SE for three independent experiments. For each treatment, at least 60 stomatal apertures were measured and data presented are the means of at least 3 independent experiments (2 leaves per experiment).
