# Supplementary figures and images for: Ectopic Expression of PII Induces Stomatal Closure in Lotus japonicus
Source: Front Plant Sci. 2017 Jul 25;8:1299. doi: 10.3389/fpls.2017.01299 (PMC5524832; doi:10.3389/fpls.2017.01299)

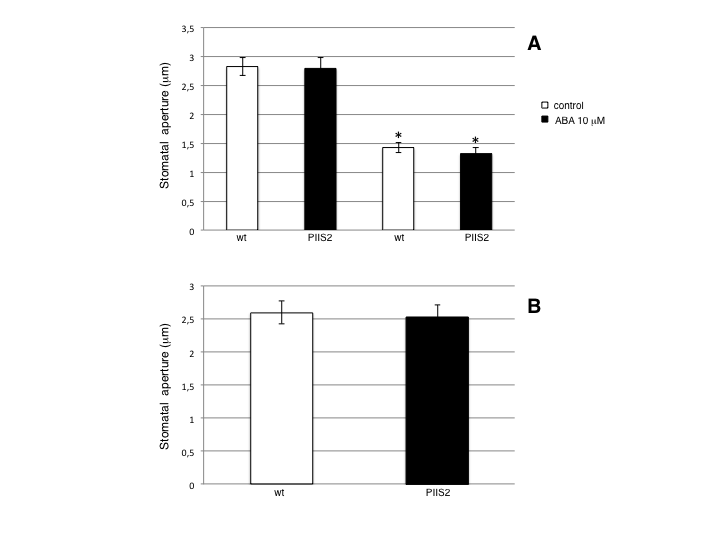

Supplement: Figure S2 — Analysis of stomata aperture in A. thaliana Columbia ecotype and PIIS2 plants. (A) Effect of 10 μM ABA on stomata closure. Epidermal strips from detached leaves were exposed to light for 3 h and then treated with (black bars) and without ABA (white bars) for an additional hour. Asterisks indicate significant differences between ABA-treated and un-treated plants (p < 0.0001; VassarStats analysis of variance program). (B) Stomata aperture in detached leaves isolated at the 6th hour of the daily light period. Columbia wild type and PIIS2 plants are indicated. Bars represent the average and SE for three independent experiments. For each treatment, at least 60 stomatal apertures were measured and data presented are the means of at least 3 independent experiments (2 leaves per experiment). [file Image2.TIFF]
